# Development and Validation of a Deep Learning Algorithm to Differentiate Colon Carcinoma From Acute Diverticulitis in Computed Tomography Images

**DOI:** 10.1001/jamanetworkopen.2022.53370

**Published:** 2023-01-27

**Authors:** Sebastian Ziegelmayer, Stefan Reischl, Hannah Havrda, Joshua Gawlitza, Markus Graf, Nicolas Lenhart, Nadja Nehls, Tristan Lemke, Dirk Wilhelm, Fabian Lohöfer, Egon Burian, Philipp-Alexander Neumann, Marcus Makowski, Rickmer Braren

**Affiliations:** 1 Institute of Diagnostic and Interventional Radiology, Technical University of Munich, School of Medicine, Munich, Germany; 2 Department of Surgery, Technical University of Munich, School of Medicine, Munich, Germany

## Abstract

**Importance:**

Differentiating between malignant and benign etiology in large-bowel wall thickening on computed tomography (CT) images can be a challenging task. Artificial intelligence (AI) support systems can improve the diagnostic accuracy of radiologists, as shown for a variety of imaging tasks. Improvements in diagnostic performance, in particular the reduction of false-negative findings, may be useful in patient care.

**Objective:**

To develop and evaluate a deep learning algorithm able to differentiate colon carcinoma (CC) and acute diverticulitis (AD) on CT images and analyze the impact of the AI-support system in a reader study.

**Design, Setting, and Participants:**

In this diagnostic study, patients who underwent surgery between July 1, 2005, and October 1, 2020, for CC or AD were included. Three-dimensional (3-D) bounding boxes including the diseased bowel segment and surrounding mesentery were manually delineated and used to develop a 3-D convolutional neural network (CNN). A reader study with 10 observers of different experience levels was conducted. Readers were asked to classify the testing cohort under reading room conditions, first without and then with algorithmic support.

**Main Outcomes and Measures:**

To evaluate the diagnostic performance, sensitivity, specificity, positive predictive value (PPV), and negative predictive value (NPV) were calculated for all readers and reader groups with and without AI support. Metrics were compared using the McNemar test and relative and absolute predictive value comparisons.

**Results:**

A total of 585 patients (AD: n = 267, CC: n = 318; mean [SD] age, 63.2 [13.4] years; 341 men [58.3%]) were included. The 3-D CNN reached a sensitivity of 83.3% (95% CI, 70.0%-96.6%) and specificity of 86.6% (95% CI, 74.5%-98.8%) for the test set, compared with the mean reader sensitivity of 77.6% (95% CI, 72.9%-82.3%) and specificity of 81.6% (95% CI, 77.2%-86.1%). The combined group of readers improved significantly with AI support from a sensitivity of 77.6% to 85.6% (95% CI, 81.3%-89.3%; *P* < .001) and a specificity of 81.6% to 91.3% (95% CI, 88.1%-94.5%; *P* < .001). Artificial intelligence support significantly reduced the number of false-negative and false-positive findings (NPV from 78.5% to 86.4% and PPV from 80.9% to 90.8%; *P* < .001).

**Conclusions and Relevance:**

The findings of this study suggest that a deep learning model able to distinguish CC and AD in CT images as a support system may significantly improve the diagnostic performance of radiologists, which may improve patient care.

## Key Points

**Question:**

Can a deep learning algorithm differentiate between acute diverticulitis and colon cancer on computed tomography images and improve radiologists’ performance under routine clinical conditions?

**Findings:**

In this diagnostic study, a 3-dimensional convolutional neural network developed on contrast-enhanced computed tomography images of 585 patients with colon cancer and acute diverticulitis was able to predict both entities with a high sensitivity (83%) and specificity (87%). As an artificial intelligence support system, the model significantly improved the sensitivity and specificity and reduced the number of false-negative and false-positive findings.

**Meaning:**

The findings of this study suggest that, as a support system, a deep learning model may improve the care of patients with large-bowel wall thickening.

## Teaser

This diagnostic study evaluates a deep learning algorithm developed to distinguish colon carcinoma from acute diverticulitis.

## Introduction

Acute diverticulitis (AD) is a frequent gastrointestinal cause for hospital admission with a substantial disease burden.^1^ In particular, less typical forms of presentation, such as right-sided localization or young patient age, can obfuscate and delay the correct diagnosis. Contrast-enhanced computed tomography (CT) is the imaging modality of choice, and imaging signs include bowel wall thickening, fat stranding, enlarged local lymph nodes, and the presence of diverticula, none of which is specific to AD.^2,3^ However, radiologic differentiation from its most important differential diagnosis, colon cancer (CC), remains difficult due to an overlap of imaging features. Prior studies have reported a radiologic sensitivity and specificity in the differentiation of CC (40%-95.5%) and AD (66%-93.3%).^3,4^ However, upper margin values are often not reached in clinical practice scenarios.

The management of complicated AD vs CC may vary substantially with minimal damage control surgery as a viable option in patients who are frail and older with complicated AD compared with oncologic surgery required in cases of CC. Furthermore, episodes of AD require follow-up colonoscopy to exclude cancer. Prevalence of CC at follow-up colonoscopy after AD ranges widely and has been reported to be up to 40-fold increased.^5,6^ A probably more realistic meta-analysis by Meyer et al^7^ reported a 2-fold increased risk of carcinoma for uncomplicated and complicated diverticulitis in comparison with the general population.^7,8^ Colonoscopy is an invasive examination with a nonnegligible risk of complications^9,10^ and should therefore be avoided if possible. In addition, colonoscopy should be performed only after the acute episode to mitigate complications, which can delay the diagnosis of an underlying CC. Improved radiologic accuracy could reduce the number of follow-up colonoscopies and in the emergency setting support the planning of an adequate surgical approach.^11^

The rapid development of artificial intelligence (AI) in image processing is facilitating the transfer of new methods to medical image analysis. Over the past decade, promising results have been presented for numerous tasks in medical image analysis, with deep learning algorithms being on par or even outperforming human interpretation for specified tasks, for example, in the case of breast and lung cancer detection.^12,13^ Supporting radiologic expertise with AI support systems may further improve diagnostic accuracy as shown for breast cancer detection in mammograms.^14,15^ In particular, in the primary care emergency setting where access to expert radiologist opinion may be limited, such AI support systems may increase overall radiologic accuracy and patient outcome. Therefore, the aim of our study was to develop a deep learning algorithm able to differentiate AD and CC in routinely acquired CT scans and test the algorithm as an AI support system.

## Methods

### Study Design and Patient Collective

The diagnostic study was designed as a single-center retrospective medical records study at our tertiary institution. It was approved by the ethical review board of the Technical University of Munich. Informed consent was waived according to the regulations of the university for retrospective analyses. The study was conducted in accordance with the Transparent Reporting of a Multivariable Prediction Model for Individual Prognosis or Diagnosis (TRIPOD) and the Checklist for Artificial Intelligence in Medical Imaging (CLAIM).

**Figure 1.**
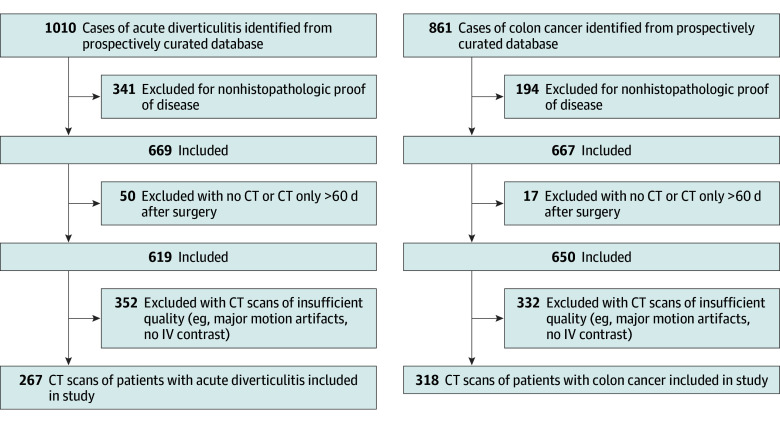
Consecutive Patient Inclusion Flowchart CT indicates computed tomography.

Patients who underwent surgery for CC or AD at our institution between July 1, 2005, and October 1, 2020, were identified in a prospectively curated database. Patient inclusion criteria were (1) histopathologic proof of diverticular disease or colonic cancer, (2) venous phase CT imaging up to 60 days preoperatively, and (3) segmental wall thickening of the colon independent of the disease stage. The only exclusion criterion was insufficient CT image quality (eg, noncontrast scans and major motion artifacts) (Figure 1; eFigure 1A in Supplement 1).

### Image Preprocessing and Model Development

**Figure 2.**
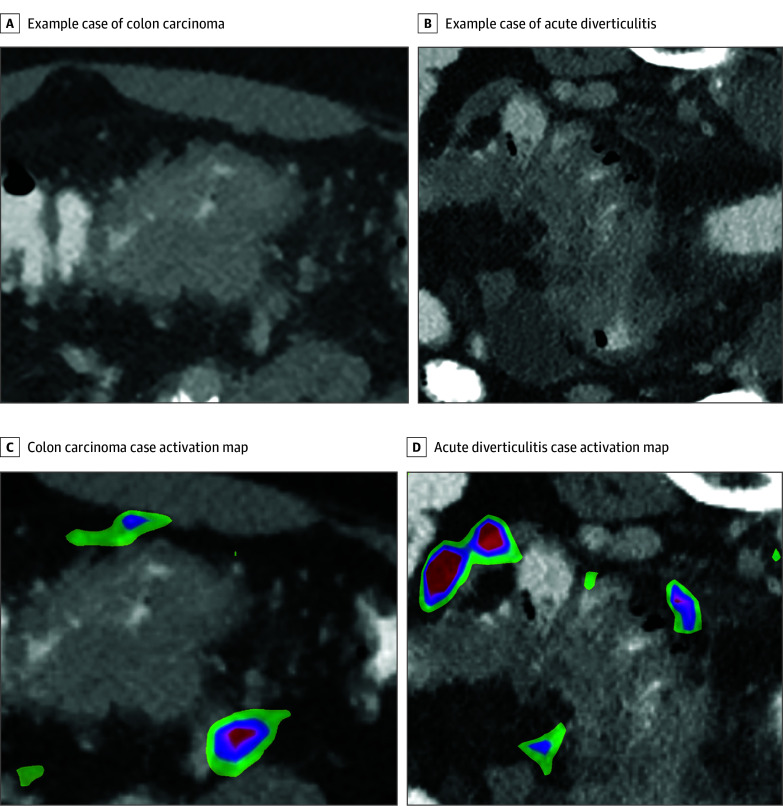
Example Cases of Colon Carcinoma and Acute Diverticulitis A, Example of colon carcinoma with a short segment wall thickening, no pericolic stranding, and mesenterial tumor extension. B, Example of acute diverticulitis with a long segment of wall thickening, pericolic fluid and stranding, and diverticula. C, Tumor extension in the right lower quadrant in colon carcinoma. D, Pericolic fat strands and fluid collection show the highest activity in acute diverticulitis. The wall thickening itself shows no activation. Colored areas indicating the respective class are displayed. The magnitude of the class score changes from red to blue.

The data set was split into training (435 [74.4%]), validation (90 [15.4%]), and testing (60 [10.2%]) cohorts. For the test set, AD and CC cases were balanced. All patient data were exported in deidentified form. For each CT scan, a 3-dimensional (3-D) bounding box with fixed dimensions of 128 × 128 × 24 pixels was cropped around the center of the pathologic imaging finding by a trained observer using ITK-snap, 3.8.0.^16^ Each box was reevaluated and corrected, if necessary, in consultation with an experienced abdominal radiologist. Bounding boxes included bowel wall thickening, pericolonic fat/adjacent mesentery, and, if present, local lymph nodes. Example boxes are shown in Figure 2A and B. Image preprocessing, model development, training, and evaluation were done using Python, version 3.9 (Python Software Foundation) and open source TensorFlow, version 2.4. Bounding boxes were normalized and augmented by random rotation. Model architecture, parameters, and training schedule can be found in the eMethods in Supplement 1. In brief, an 18-layer 3-D CNN with a batch size of 16 and an input tensor shape of 128 × 128 × 24 × 1 pixels was trained for 120 epochs and the best model parameters were retained based on validation accuracy. The model was evaluated on the test set. Sensitivity, specificity, negative predictive value (NPV), positive predictive value (PPV), false-negative rate, and false-positive rate were calculated. Gradient-weighted class activation mapping adapted from Selvaraju et al^17^ was used to generate activation maps for both classes. Sensitivity analysis was performed using random rotation and additive noise (eMethods in Supplement 1) The model was externally validated on 126 colon cancer cases from the open-source medical segmentation decathlon data set (eMethods in Supplement 1).

### Reader Study Protocol

Ten readers of different expertise levels were included in the study, while 1 author (S.Z.) supervised the reader study. The reader distribution was as follows: radiology residents with less than 3 years of experience (n = 3), radiology residents with 3 or more years of experience (n = 4), and board-certified radiologists (n = 3), with 2 having a specialization in gastrointestinal imaging. Full-field axial CT images from the test set were presented to the readers. To exclude bias with respect to metastasis, the liver was excluded from the volumes. Images were deidentified and presented blinded in random order under reading room conditions without further clinical information. Readers were asked to sequentially classify each case as CC or AD. After the initial classification for the individual case (diagnosis without AI support), readers were presented with the prediction of the algorithm (probability for each class) and were allowed to change or keep their initial assessment for the present case (diagnosis with AI support). The model sensitivity and specificity were not revealed to the observer prior to the reading, and readers did not receive any feedback on the correctness of the diagnosis. Observers were split into 2 groups (resident and board-certified radiologists). Sensitivity, specificity, NPV, PPV, false-negative rate, and false-positive rate were calculated for both reader groups. Statistical analysis was performed in R, version 3.6.3 (R Foundation for Statistical Computing). Metrics for all readers and the subcohorts were compared using the McNemar test for sensitivity and specificity and relative and absolute predictive value comparisons for PPV and NPV. A 2-sided, paired *P* value <.05 was considered significant.

## Results

### Patient Collective

A total of 585 patients (mean [SD] age, 63.2 [13.4] years; 341 men [58.3%], and 244 women [41.7%]) were included in the analysis. All included patients had bowel resections of the diseased bowel with histopathologic confirmation of the diagnosis by a board-certified pathologist. No patients had missing data on included parameters. Patients with CC (n = 318) had a mean (SD) age of 66.6 (13.2) years and 189 were men (59.4%). Patients with AD (n = 267) had a mean (SD) age of 59.0 (12.5) years and 152 were men (56.9%). Of patients with AD, 30 had staging, with the American Joint Committee on Cancer classification of diverticular disease (CDD), of 1a; 114 were CDD 1b; 29 were CDD 2a; 78 were CDD 2b; and 16 were CDD 2c. Of patients with CC, 11 were Union for International Cancer Control (UICC) 0, 50 were UICC I, 84 were UICC II, 88 were UICC III, and 85 were UICC IV. Patient characteristics and stages of disease are displayed in Table 1 for both entities. Computed tomographic scanner and contrast protocols are listed in the eTable in Supplement 1. Of 585 patients, 445 (77.8%) underwent the scan internally and 130 (22.8%) patients received external imaging.

**Table 1.** Stage of Disease in the Included Patient Collective

Variable	Total	Diverticulitis (n = 267)	Colorectal carcinoma (n = 318)
Age, mean (SD), y	63.2 (13.4)	59.0 (12.5)	66.6 (13.2)
Sex, No. (%)			
Female	244 (41.7)	115 (43.0)	129 (40.6)
Male	341 (58.3)	152 (56.9)	189 (59.4)
Classification, No. (%)			
CDD 1a	NA	30 (11.2)	NA
CDD 1b	NA	114 (42.7)	NA
CDD 2a	NA	29 (10.9)	NA
CDD 2b	NA	78 (29.2)	NA
CDD 2c	NA	16 (6.0)	NA
UICC 0	NA	NA	11 (3.5)
UICC I	NA	NA	50 (15.7)
UICC II	NA	NA	84 (26.4)
UICC III	NA	NA	88 (27.7)
UICC IV	NA	NA	85 (26.7)

Abbreviations: CDD, classification of diverticular disease; NA, not applicable; UICC, Union for International Cancer Control.

### Stand-alone AI System Performance

**Figure 3.**
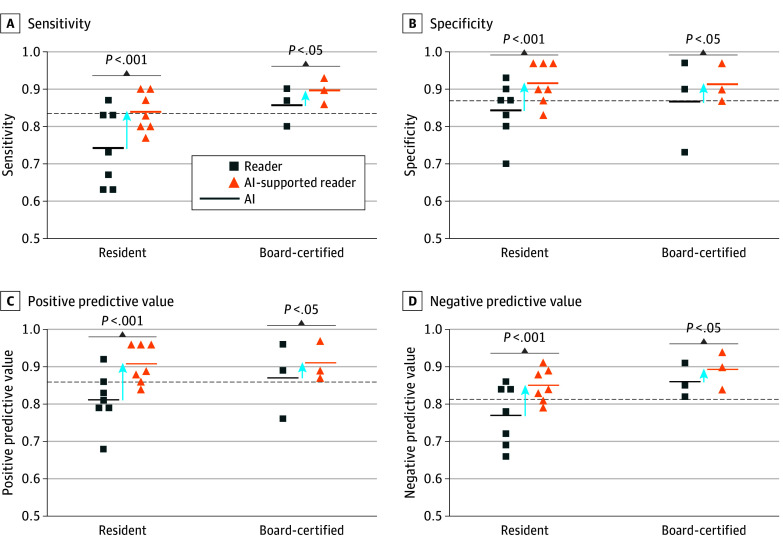
Stand-alone Performance of Artificial Intelligence (AI) Sensitivity (A), specificity (B), positive predictive values (C), and negative predictive values (D) relating to colon carcinoma for both reader groups without and with AI support. The AI dotted lines indicate the stand-alone AI performance.

At a decision threshold of 0.5, the stand-alone AI support system reached a sensitivity and specificity of 98% and 92% for the training set and 94% and 83.3% for the validation set. For the test set model, sensitivity was 83.3% (95% CI, 70.0%-96.6%) and specificity was 86.6% (95% CI, 74.5%-98.8%), comparable to the performance of the board-certified reader group, with a sensitivity of 85.5% (95% CI, 78%-93%) and specificity of 86.6% (95% CI, 80%-94%). The NPV was 83.8% (95% CI, 70.9%-96.8%) and the PPV was 86.2% (95% CI, 73.6%-98.7%). The AI support system showed a false-negative rate of 16% and a false-positive rate of 13% (eFigure 1C in Supplement 1) (Figure 3). For the Medical Segmentation Decathlon (MSD) data set, the model reached an accuracy of 88.8%. The results of the sensitivity analysis can be found in the eMethods of Supplement 1. In short, image rotation showed no to marginal effect and additive noise had a substantial impact on model performance at a threshold of 0.01 variance. The corresponding images with the additive noise are shown in eFigure 2 in Supplement 1.

### Class Activation Maps

Exemplary activation maps for colon carcinoma and diverticulitis can be found in Figure 2C and D. Activation patterns for the example cases are primarily located pericolic, whereas bowel wall thickening showed no activation. For the CC cases, activation is displayed on the extracolonic tumor extension, whereas for the AD cases, activation can be found primarily on fluid collections and pericolic fat stranding.

### Reader Performance

The diagnostic performance of all readers (sensitivity, 77.6%; 95% CI, 72.9%-82.3%; specificity, 81.6%; 95% CI, 77.2%-86.1%; PPV, 80.9%; 95% CI, 76.3%-85.4%; and NPV, 78.5%; 95% CI, 73.9%-83.0%) was comparable to the stand-alone deep learning model (sensitivity, 83.3%; 95% CI, 70.0%-96.6%; specificity, 86.6%; 95% CI, 74.5%-98.8%; PPV, 86.2%; 95% CI, 73.6%-98.7%; and NPV, 83.8%; 95% CI, 70.9%-96.8%). The unsupported performance of the 2 reader groups (resident and board-certified) is presented in Table 2. The overall accuracy for the reader groups was 79.2% for the residents and 86.1% for the board-certified radiologists. Diagnostic performance of readers depended on their experience level. As expected, board-certified radiologists performed better than radiology residents; notably, sensitivity improved by 11.3%. Using the model as a support system showed a significant increase in sensitivity, specificity, PPV, and NPV for all readers and both subgroups. The combined reader group showed a significant increase in sensitivity (85.6%; 95% CI, 81.3%-89.3%; *P* < .001), specificity (91.3%; 95% CI, 88.1%-94.5%; *P* < .001), PPV (90.8%; 95% CI, 87.4%-94.1%; *P* < .001), and NPV (86.4%; 95% CI, 82.3%-89.9%; *P* < .001). Artificial intelligence–supported performance for both reader groups is listed in Table 2. For the radiology residents, sensitivity improved by 9.6% (*P* < .001), specificity by 7.2% (*P* < .001), PPV by 8.2% (*P* < .001), and NPV by 8.3% (*P* < .001). For the board-certified radiologists, sensitivity improved by 4.5% (*P* = .045), specificity by 4.7% (*P* = .045), PPV by 4.6% (*P* = .03), and NPV by 4.3% (*P* = .03). Due to the divergent management of patients with CC, the reduction of false-negative findings is of utmost importance. Without an AI support system, the false-negative rate was 22% for all readers, 26% for the residents, and 14% for the board-certified radiologists. Artificial intelligence support led to substantial reduction in the false-negative rate to 14.3% for the combined reader group, 16.1% for the residents, and 10.0% for the board-certified radiologists. Artificial intelligence support prompting readers to switch from a true-positive to false-negative only occurred 4 times (0.6%), of which 3 were made by residents.

**Table 2.** Diagnostic Performance of Residents and Board-Certified Radiologists Without and With AI Support

Variable	% (95% CI)			
	Resident	Resident + AI	Board-certified	Board-certified + AI
Sensitivity	74.2 (68-80)	83.8 (78-88)	85.5 (78-93)	90.0 (84-96)
Specificity	84.2 (79-89)	91.4 (87-95)	86.6 (80-94)	91.3 (85-97)
PPV	82.5 (79-89)	90.7 (87-95)	86.5 (79-93)	91.1 (85-97)
NPV	76.6 (71-82)	84.9 (80-90)	85.7 (79-93)	90.0 (84-96)

Abbreviations: AI, artificial intelligence; NPV, negative predictive value; PPV, positive predictive value.

## Discussion

In our study, we aimed to address the remaining radiologic problem to differentiate between benign and malignant etiology of bowel wall thickening. In this study, we developed a 3-D CNN as an AI support system for radiologists to improve their diagnostic performance in the CT image-based separation of CC and AD.

The AI model showed noninferior performance to the average reader and led to a significant increase in the reader performance independent of the reader experience. Furthermore, AI support resulted in a decrease of false-negative rates in both reader groups. Class activation maps revealed activation patterns of the algorithm focusing on classical secondary imaging findings.

The correct differentiation of CC and AD has major clinical implications. In the perforated stage, both entities require emergency surgery; however, the surgical strategies differ. Whereas CC requires oncologic resection of the diseased bowel and the entire lymph node basin, a limited resection of the diseased bowel may suffice in cases of AD. A high level of certainty in surgical planning improves patient stratification and thus limits postoperative complications and potentially decreases mortality rates.^18^ Particularly in cases of emergency surgery and in limited resource settings, a high level of diagnostic accuracy is indispensable. Even in the nonperforated stage, a precise classification of the diseases is important for patient triage. Current guidelines recommend follow-up colonoscopy for patients with AD to exclude malignant disease, which could be avoided in cases of sufficient certainty for nonmalignant disease.^19^

The purely morphologic differentiation of AD from CC based on CT imaging remains difficult. In uncomplicated AD and early-stage CC, subtle changes in CT imaging, such as focal bowel wall thickening and adjacent fat stranding, present the only finding and may be obscured by bowel filling, especially in the emergency setting without proper patient preparation, or mistaken for peristaltic activity when concentric in appearance. In complicated AD and advanced-stage CC, secondary changes, such as mesenteric stranding, free fluid and abscess formation as a result of major inflammation or long-lasting obstruction, can become the dominating imaging features. Prior studies attribute radiologic signs, such as mesenteric fluid, fat stranding, and abscess formation, to AD, whereas focal mass formation and pathologic lymph nodes are ascribed to CC.^2,3,20^ However, the only prospective study on CT image-based differentiation of AD and CC reached a correct diagnosis in just 49% of the cases,^4^ indicating major overlap of the imaging features. In our study, diagnostic accuracy was dependent on the readers’ level of experience, ranging from 79.2% to 86.1%.

The potential benefit of AI in gastrointestinal imaging has been demonstrated by several studies^21,22,23^; however, for CC, mostly histopathologic and endoscopic models exist.^24^ In particular, to our knowledge, the differentiation of CC and AD in CT scans by methods of AI has not been investigated so far. The stand-alone model showed noninferiority in comparison with the board-certified readers with a sensitivity of 83.3% (95% CI, 70.0%-96.6%) vs 85.5% (95% CI, 78%-93%) and specificity of 86.6% (95% CI, 74.5%-98.8%) vs 86.6% (95% CI, 80%-94%). While models using the entire CT volume as input are desirable, this approach was not feasible due to the limited number of patients. Therefore, 3-D bounding boxes including the affected bowel segment were used as input (eFigure 1B in Supplement 1). A potential clinical application of our model requires a user interface in which the radiologist can manually define the affected bowel segment.

The reader study was conducted to simulate the use of an AI-based support system on an individual case level in reading room conditions, to observe the utility of support applications in the characterization of large-bowel wall thickening of the colon. Our model significantly increased the diagnostic performance of all readers, proving the feasibility of AI-supported image analysis. Similar results were shown for the use of an AI model as a second reader in mammographies, where studies indicate improved diagnostic accuracy and significant reduction in reading time.^14,25^ In addition, AI support led to a reduction in the false-negative rate, which may provide a more accurate diagnostic and therapeutic approach. The relative reduction of false-negative rates for CC by AI support was substantial for the overall reader and both reader groups. The number of false-negative findings provoked by AI support for CC cases was low (4 of 600 decisions [0.6%]); notably, only 1 switch was made by a board-certified radiologist.

Lastly, explanation and generalizability are general concerns in the stepwise introduction of AI support systems. We used class activation maps to visualize discriminative image regions used by the CNN to identify a specific class in the image. As shown in the attention heatmap (Figure 2C and D), the AI support system was activated by changes in pericolonic fat tissue, including regions of increased focal attenuation and fluid collections. In contrast, bowel wall thickening itself did not activate the CNN. In analogy to the radiologist’s diagnostic approach, bowel wall thickening guides the radiologist's attention in the full-field CT scan to a specific region and the detailed analysis of the pericolonic fat alterations enables the final diagnosis. In addition, we evaluated the model on unseen colon cancer cases from the MSD data set, where the model achieved 88.8% accuracy.

### Limitations

This study has limitations. Foremost, our model was trained and tested on a single institutional data set, and therefore may not reach broader generalization. However, we included 22.8% (n = 130) of patients whose scans were performed externally to generate CT scanner and contrast protocol heterogeneity (eTable in Supplement 1). Moreover, we validated the model on the MSD data set and were able to show good accuracy for CC cases. Unfortunately, no external cases of AD were available. Although the model was robust to anatomic variation simulated by rotation, adversarial noise at a variance threshold of 0.01 had a substantial impact on model performance. It can be assumed that relevant image features, for example, fat stranding, are masked at this noise level (eFigure 2 in Supplement 1). Adapted model training to reduce the impact of potential adversarial noise is necessary. The reader study simulated the use of an AI-based support system on an individual case level for the test set in reading room conditions. While this allows a rough analysis of the usability of the support system in a clinical setting, follow-up studies are needed to simulate a more realistic integration, with respect to the distribution of findings. In this proof-of-concept study we only included the most frequent malignant and benign entities for bowel wall thickening; in further studies the model should be adapted for malignant and benign entities in general. Furthermore, multiparametric data integration, including laboratory inflammatory markers, vital signs, and other symptoms, could improve the model and should be included in further projects.

## Conclusions

In this diagnostic study, we developed a 3-D CNN that can be implemented as an AI support system for the differentiation of CC and AD based on CT images. Artificial intelligence support led to a significant increase in diagnostic performance of board-certified radiologists and radiology residents.

## Notes

Supplement 1. **eMethods.** Detailed Methods **eTable.** CT Scanner and Contrast Protocol Listing **eFigure 1.** Study Design **eFigure 2.** Exemplary Images With Additive Gaussian Noise and the Respective Model Performance

Supplement 2.
Data Sharing Statement
