# Supplementary material for: Development and Validation of a Deep Learning Algorithm to Differentiate Colon Carcinoma From Acute Diverticulitis in Computed Tomography Images
Source: JAMA Netw Open. 2023 Jan 27;6(1):e2253370. doi: 10.1001/jamanetworkopen.2022.53370 (PMC11984516; doi:10.1001/jamanetworkopen.2022.53370)
Supplement: Supplement 1. — eMethods. Detailed Methods eTable. CT Scanner and Contrast Protocol Listing eFigure 1. Study Design eFigure 2. Exemplary Images With Additive Gaussian Noise and the Respective Model Performance [file jamanetwopen-e2253370-s001.pdf]

Supplementary Online Content

Ziegelmayer S, Reischl S, Havrda H, et al. Development and validation of a deep learning algorithm to differentiate colon carcinoma from acute diverticulitis in computed tomography images. *JAMA Netw Open*. 2023;6(1):e2253370. doi:10.1001/jamanetworkopen.2022.53370

eMethods. Detailed Methods

eTable. CT Scanner and Contrast Protocol Listing

eFigure 1. Study Design

eFigure 2. Exemplary Images With Additive Gaussian Noise and the Respective Model Performance

This supplementary material has been provided by the authors to give readers additional information about their work.

© 2023 Ziegelmayer S et al. *JAMA Network Open.*

eMethods. Detailed Methods

Model description

An 18-layer 3D-CNN architecture was used, with 3 x 3 x 3 kernel size for the convolutional layers and max pooling and batch normalization layers after each convolution. The input volume was defined based on the bounding box dimensions resulting in a 3-dimensional input tensors of shape batch size × 128 × 128 × 24 × 1. The model was randomly initialized and trained with a batch size of 16 and binary cross entropy as the loss function. The learning rate was set to 0.0001 with an exponential decay learning rate schedule of 0.96 every 100000 steps. Adam was used as an optimizer. The model was trained for 120 epochs and the best model parameters were retained based on validation accuracy.

External model validation on the MSD dataset.

The trained model was evaluated on the open source medical segmentation decathlon dataset (cite) . The dataset includes 190 cases of CT-scans from therapy naive colon cancer patients. The training dataset included 126 patients with segmentation masks of the bowel wall thickening. 3D bounding boxes were generated using PyTorch and the package TorchIo. A target volume with shape 128 x 128 x 24 was cropped around the segmentation mask. The bounding boxes were preprocessed in similar fashion to the internal test set. The model reached an accuracy of 88,8% classifying 112 of 126 cases correctly.

Model Sensitivity analysis.

A sensitivity analysis with stepwise image transformation was conducted on the test set. Firstly, images were randomly rotated in between 0 to 360°. Secondly images were transformed using gaussian distributed additive noise. The variance for the random labels was exponentially increased from 0.0001 to 0.1. Random image rotation led to one classification change with a corresponding sensitivity and specificity of 83.3% and 83.3%. Gaussian noise affected model started to substantially affect model performance at a variance of 0.01. Exemplary images of the varying noise with the respective model performance are shown in eFigure2.

© 2023 Ziegelmayer S et al. *JAMA Network Open.*

eTable CT-scanner and contrast protocol listing

**Siemens**	
Siemens SOMATOM Definition AS	146
Siemens Sensation Cardiac 64	105
Siemens Emotion	33
Siemens Sensation 16	31
Siemens Volume Zoom	5
Siemens Biograph	2
Siemens Balance	1
**Philips**	
Philips IQon – Spectral CT	112
Philips iCT 256	92
Philips Brilliance	22
Philips Ingenuity CT	6
Philips Mx8000 IDT 16	4
**Toshiba**	
Toshiba Aquilion	10
Toshiba Activion	3
Toshiba Astelion	1
**GE MEDICAL**	
GE MEDICAL SYSTEM LightSpeed	8
GE MEDICAL SYSTEM Optima CT660	3
GE MEDICAL SYSTEM Revolution HD	1

Contrast agent	
Intravenous contrast	52
Intranveous and positive oral contrast	97
Intranveous and positive rectal contrast	271
Intranveous and positive oral and rectal contrast	165

© 2023 Ziegelmayer S et al. *JAMA Network Open.*

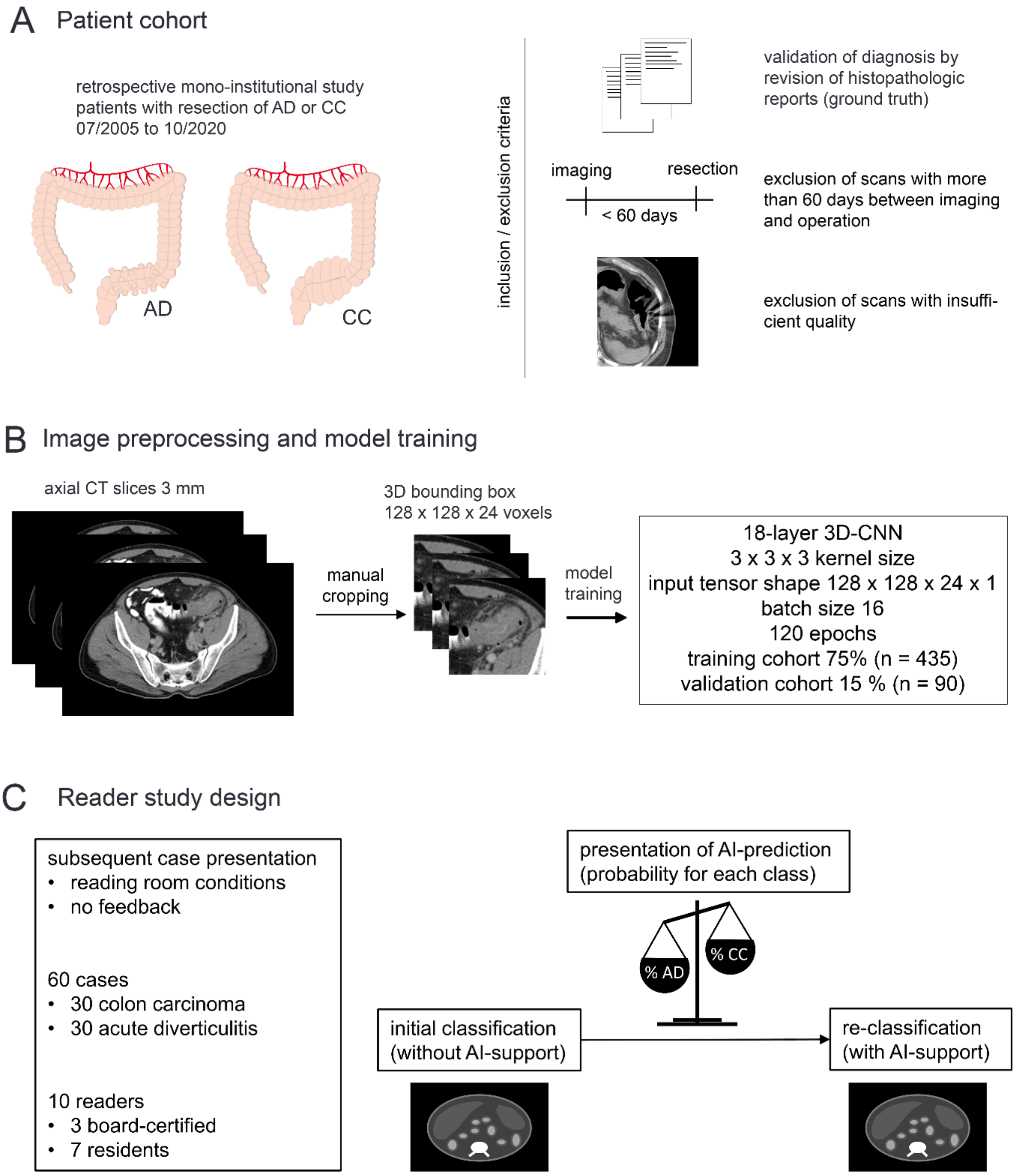

eFigure1 Study design
(A) All patients operated for colon carcinoma (CC) or acute diverticulitis (AD) between 2005 and 2020 were determined. Diagnosis was verified by detailed review of histopathological reports of the specimens. Only patients with less than 60 days between CT imaging and surgery were included. Patients with insufficient CT quality (e.g. non-contrast images, major motion artifacts) were excluded. (B) Axial CT images were cropped manually to standardized 3D-bounding boxes. A 3D-CNN was trained on 75% of cases and validated on 15 % of cases. (C) A reader study was performed including 60 cases (10 % of the cohort; 30 CC / 30 AD), which were classified by10 readers of different experience levels. After revealing the probabilities calculated by the AI-model, readers were allowed to change their decision.

© 2023 Ziegelmayer S et al. *JAMA Network Open.*

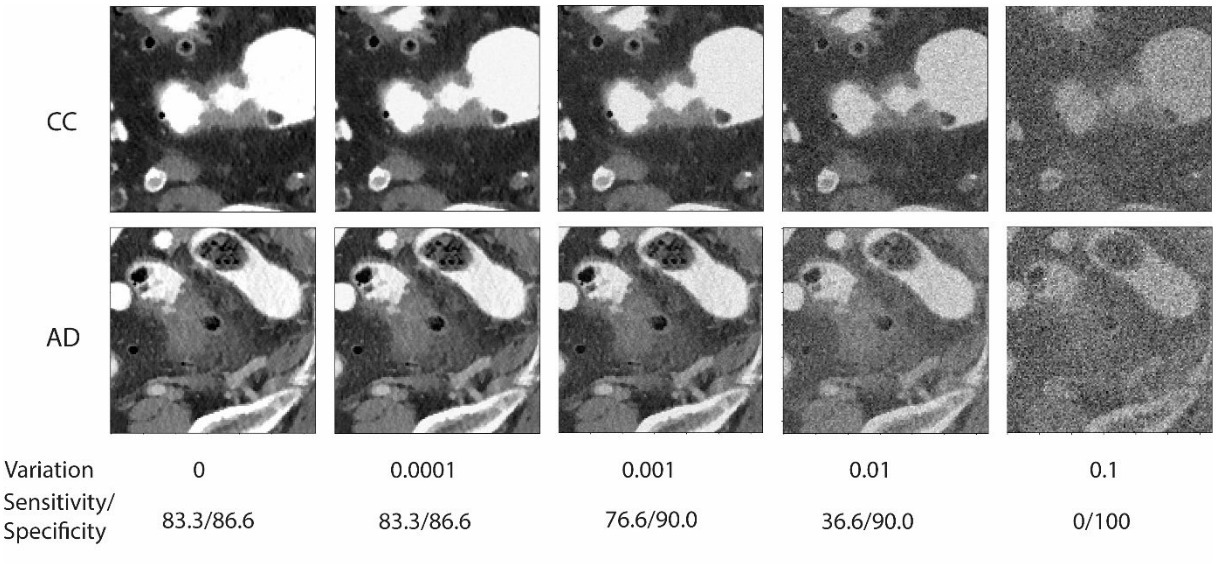

eFigure 2 Exemplary images with additive gaussian noise and the respective model performance

© 2023 Ziegelmayer S et al. *JAMA Network Open.*
