# Supplementary material for: Development and Validation of a Deep Learning Algorithm to Differentiate Colon Carcinoma From Acute Diverticulitis in Computed Tomography Images
Source: JAMA Netw Open. 2023 Jan 27;6(1):e2253370. doi: 10.1001/jamanetworkopen.2022.53370 (PMC11984516; doi:10.1001/jamanetworkopen.2022.53370)
Supplement: Supplement 2. — Data Sharing Statement [file jamanetwopen-e2253370-s002.pdf]

Data Sharing Statement

Ziegelmayer. Development and Validation of a Deep Learning Algorithm to Differentiate Colon Carcinoma From Acute Diverticulitis in Computed Tomography Images. *JAMA Netw Open*. Published January 27, 2023. doi:10.1001/jamanetworkopen.2022.53370

Data
Data available: No
